# Regional prevalence and determinants of exclusive breastfeeding in India

**DOI:** 10.1186/s13006-019-0214-0

**Published:** 2019-05-16

**Authors:** Felix Akpojene Ogbo, Mansi Vijaybhai Dhami, Akorede O. Awosemo, Bolajoko O. Olusanya, Jacob Olusanya, Uchechukwu L. Osuagwu, Pramesh Raj Ghimire, Andrew Page, Kingsley E. Agho

**Affiliations:** 1 Translational Health Research Institute (THRI), School of Medicine, Western Sydney University, Campbelltown Campus, Locked Bag 1797, Penrith, NSW 2571 Australia; 2 Prescot Specialist Medical Centre, Welfare Quarters, Makurdi, Benue State Nigeria; 3 Centre for Healthy Start Initiative, 286A Corporation Drive, Dolphin Estate, Ikoyi, Lagos, Nigeria; 4 School of Medicine | Diabetes Obesity and Metabolism Translational Research Unit (DOMTRU), Macarthur Clinical School, Parkside Crescent, Campbelltown, NSW 2560 Australia; 5 School of Science and Health, Western Sydney University, Campbelltown Campus, Locked Bag 1797, Penrith, NSW 2571 Australia

**Keywords:** Exclusive breastfeeding, Infants, India, Region, Nutrition

## Abstract

**Background:**

Exclusive breastfeeding (EBF) has important benefits for both the mother and child. In India, no nationwide studies have examined patterns of EBF in the past decade to inform national and subnational breastfeeding programmes. The present study aimed to investigate the regional prevalence and determinants of EBF in India.

**Methods:**

This study used a total weighted sample of 21,352 from the 2015–2016 India National Family Health Survey. EBF was measured as the proportion of infants 0–5 months of age who received breast milk as the only source of nourishment, based on mother’s recall on feeds given to the infant 24 h before the survey. The prevalence of EBF and other breastfeeding patterns were estimated by region, and multivariable logistic regression that adjusted for clustering and sampling weights was used to investigate the association between the study factors (child, maternal, household, health service and community factors) and EBF by regional areas in India.

**Results:**

This study indicated that wide differences in the prevalence of EBF and other childhood feeding practices exist across regions of India, where Southern India had the highest EBF prevalence (79.2%) and the North-East reported the lowest (68.0%). EBF prevalence decreased with infant age, dropping faster in the South (43.7% at 5 months) compared to the North-East region (54.0% at 5 months). Similarly, substantial variations in key determinants of EBF were evident by region, where higher birth order was the only common factor associated with non-EBF across all regions. Key modifiable determinants of non-EBF included higher maternal education in the South and belonging to rich households in Central India, while those for EBF were higher maternal education in the Central region and frequent antenatal care (≥ 4) visits in Northern India.

**Conclusion:**

This study demonstrates wide variations in regional prevalence and determinants of EBF in India. Improving EBF participation in India would require multifaceted national and subnational efforts that include dedicated funds and the establishment of appropriate policy and interventions that are consistently monitored and evaluated.

**Electronic supplementary material:**

The online version of this article (10.1186/s13006-019-0214-0) contains supplementary material, which is available to authorized users.

## Background

The first 1000 days (between conception and a child’s second birthday) provides a unique period of opportunity for optimum child growth and development and also establishes the foundations for good health across the life course [1]. During this period, appropriate infant and young child feeding practices (IYCF) are critical for the child’s health and well-being [2–4]. Essential components of IYCF practices include timely initiation of breastfeeding, exclusive breastfeeding (EBF) for the first 6 months of life and continued breastfeeding until the child is 2 years of age, including the timely introduction of appropriate complementary foods [5]. EBF (defined as the practice of only giving an infant breast milk for the first 6 months of life, with no other food or water added) is the cornerstone of optimum infant nutrition [5]. Notably, EBF reduces the risk of the infant to experience diarrhoeal diseases [2–4, 6], upper respiratory tract infections, obesity in later life, and EBF could improve the neurocognitive functions of the child [4].

Globally, India has the highest under-five mortality (0·9 million deaths in 2016) [7], attributable to an array of factors such as poverty, poor water and sanitation, poor healthcare access and non-EBF [8, 9]. Between 2005 and 2016, past national studies from India reported an improvement in EBF prevalence by 9.0% (from 46.0 to 55.0%) [10]. However, national data often mask significant variations across the regions. For example, findings from discrete sub-national studies have shown that EBF varied widely in India, ranging from 36.0% in Meghalaya to 77.0% in Chhattisgarh [11]. Similarly, studies from informal settlements have also reported broader and lower findings, between 11.4% [12] and 63.0% [13].

A national study based on the analysis of the 1992 and 2006 India Demographic and Health Surveys (DHS) indicated that the differences in EBF prevalence may be due to the impact of sociodemographic (higher maternal education, low household wealth status and older maternal age, ≥35 years), health service (≥4 antenatal care visits) and community (urban residence) factors [14]. However, it is unclear whether these key factors have changed in the past decade given the economic and social mobility of women [15], as well as gender role differentiation in India [16, 17]. Additionally, findings from the 2015–16 India DHS – the data source for the present study – may not be comparable to previous national surveys because of differences in the sample size. In the 2006 India DHS, approximately 110,000 households were sampled from 1028 million people using the 2001 census frame [18] compared to the 2015–16 DHS, where approximately 572,000 households were sampled from 1210.2 million people based on the 2011 census frame. More importantly, the 2015–16 DHS methodology has been recognised as the standard for future national surveys [18], suggesting the need for baseline and up-to-date evidence on EBF to guide national and subnational policy interventions in India.

Furthermore, a detailed understanding of the regional prevalence along with the determinants of EBF is essential for decision-makers to provide locally relevant policies and resources, and for public health administrators to design targeted interventions to improve EBF in India. This study aimed to examine the prevalence and determinants of EBF in India. We also took advantage of the comprehensiveness of the data and will report on the prevalence of other breastfeeding practices (i.e., the provision of breast milk plus water, juice, milk and other liquids to children) in the context of EBF by regional areas in India.

## Methods

### Data sources

The study used data from the 2015–16 India DHS, also known as the National Family Health Survey (NFHS-4). The data were collected by the International Institute for Population Sciences (IIPS), supervised by the Ministry of Health and Family Welfare (MoHFW), Government of India, and technical assistance provided by Inner City Fund (ICF) International, Maryland, USA. Information relating to infant and young child feeding practices (including EBF), as well as socioeconomic characteristics, was collected from a nationally representative sample of women aged 15–49 years from 572,000 households. The total sample was obtained in a two-stage sampling design for both rural and urban areas in the country, with villages and census enumeration blocks as the primary sampling units (PSU), respectively. In the selected rural and urban PSU, approximately 300 households were selected and were further divided into segments of about 100–150 households. Two of these segments were randomly selected for the NFHS-4 using systematic sampling with probability proportional to segment size. Thus, an NFHS-4 cluster is either a PSU or a segment of a PSU. In the second stage of the sampling, 22 households were randomly selected from every selected rural and urban cluster using a systematic sampling method [18].

To obtain the sample of mothers who exclusively breastfed in the first six months of birth, as well as reduce the potential effect of recall bias [19], we restricted our analyses to the youngest living children aged 0–5 months, living with respondents (women aged 15–49 years). The total weighted sample was 21,352 maternal EBF responses. Similarly, in the assessment of other infant and young child feeding practices, the analyses were restricted to the specific age groups for those indicators, consistent with the World Health Organization (WHO) and United Nations Children’s Funds (UNICEF) definitions [5]. The survey had high response rates that varied between Indian states and territories, from 94.0% in Andhra Pradesh and West Bengal [20, 21] to 99.6% in Bihar [22]. Additional information on the survey methodology is provided in the final India DHS reports [18, 20, 22].

India is a federal union that comprises 29 states and 7 union territories ─ a total of 36 jurisdictional entities. The states and union territories are aggregated into six zonal councils to facilitate better economic integration, resource allocation and inter-state cooperation [23, 24]. In the present study, we used the six zonal regions, which include North, South, East, West, Central and North-Eastern India. The Northern region (*n* = 2731 maternal EBF responses) consists of the states of Jammu and Kashmir, Himachal Pradesh, Haryana, Delhi, Chandigarh, Punjab and Rajasthan. The Southern region (*n* = 3641) consists of the states of Andhra Pradesh, Karnataka, Kerala, Tamil Nadu, Telangana, Andaman and Nicobar Islands, Lakshadweep Islands and the Union Territory of Puducherry. The Eastern region (*n* = 5177) consists of Bihar, Jharkhand, Odisha and West Bengal. The Western region (*n* = 2572) consists of the states of Gujarat, Maharashtra, Goa, Daman and Diu as well as Dadra and Nagar Haveli. The Central region (*n* = 6425) consists of the states of Chhattisgarh, Madhya Pradesh, Uttar Pradesh and Uttarakhand. The North-Eastern region (*n* = 806) consists of the states of Arunachal Pradesh, Assam, Manipur, Meghalaya, Mizoram, Nagaland, Sikkim and Tripura.

### Outcome variable

EBF was measured as the proportion of infants 0–5 months of age who received breast milk as the only source of nourishment (but who also received oral rehydration solution, drops or syrups of vitamins and medicines). This indicator was based on mother’s recall on feeds given to the infant in the last 24 h before the survey. This was consistent with the WHO/UNICEF definitions for assessing IYCF practices [5]. The WHO /UNICEF recommend the use of the indicator, ‘predominant breastfeeding’, to measure infants 0–5 months of age who received breast milk as the main source of nourishment, but allows water, water-based drinks, fruit juice, oral rehydration solution, drops, or syrups of vitamins and medicines.

### Study factors

The study factors selected were based on evidence from previous studies [14, 25, 26]. The study factors included child, maternal, household, health service and community factors. Child factors included sex of the baby, child age, birth order, the perceived size of the baby and preceding birth interval. Maternal characteristics included the mother’s highest educational level, employment status, marital status, literacy level, religion, age and type of caste or tribe. In the DHS, a mother was classified as literate if she had completed education to standard six or higher, or was able to read a sentence or any part of the sentence from a given literacy card. A detailed description of other study factors is provided in the NFHS-4 report [18].

Household/family characteristics included the frequency of listening to the radio, the frequency of watching television and frequency of reading newspaper, and household wealth index. The household wealth index was derived from a principal components analysis conducted by the IIPS and ICF International and was calculated as a score of ownership of household assets such as transportation device, ownership of durable goods and household facilities. The IIPS and ICF International classified the household wealth index into five categories (quintiles), and each household was assigned to one of these wealth index categories namely: poorest, poorer, middle, rich and richest. These data were re-categorised, where the bottom 40% of households represent poor households, the next 40% as the middle households and the top 20% as rich households to ensure adequate sample in each category [25, 26]. Health service factors included the number of antenatal clinic visits, the place of delivery, the mode of delivery, the timing of postnatal visits and the type of delivery assistance, while one community level factor included was a place of residence.

### Statistical analysis

Initial analysis involved the estimation of frequencies and cross-tabulations to estimate the prevalence of EBF practice by region for all study factors examined. An estimation of the prevalence of EBF and other breastfeeding patterns with child age by regions was also conducted. This was followed by univariable and multivariable logistic regression generalized linear latent and mixed models (GLLAMM) with a logit link and binomial family that adjusted for clustering and sampling weights and was used to estimate the association between the study factors (child, maternal, household, health service and community factors) and EBF for each region in India.

In the multivariable analyses, a five-stage model was performed. In the first stage model, child characteristics were entered into the model and a manual stepwise elimination method was used to remove the non-significant factors (*p* > 0.05). In the second stage model, the significant factors in the first stage model were added to the maternal characteristics and this was followed by another manual stepwise elimination procedure which retained all the significant factors. A similar procedure was employed for the third and fourth stages, which included the household characteristics, as well as health services characteristics and the final stage model introduced the community factors. After completion of all five modelling stages, the factors that were significantly associated with EBF were retained.

We calculated and reported unadjusted odds ratios (Additional file 1) and statistically significant adjusted odds ratios (aORs) and their corresponding 95% confidence intervals as the measure of association between the study factors and EBF in each Indian region. The analyses were performed in Stata version 14.0 (Stata Corp, College Station, Texas, USA).

### Ethics

The DHS project sought and obtained the required ethical approvals from the Ethics Review Board of the International Institute for Population Sciences, Mumbai, India before the surveys were conducted, with informed consent obtained from participants during the surveys. Approval was sought from Measure DHS and permission was granted for this use.

## Results

### Characteristics of the study participants

Overall, the proportions of male and female children across six regions were similarly distributed, 52.8% vs 47.2% in North, 51.7% vs 48.4% in South, 52.9% vs 47.1% in East, 50.2% vs 49.8% in West, 51.4% vs 48.6% in Central, and 49.5% vs 50.6% in North-Eastern region. The prevalence of mothers who had no formal education was highest in the Eastern region (36.1%) compared to their Southern Indian counterparts (9.8%) [Table 1].

**Table 1:** Characteristics of the study population in India, 2015–16 NFHS

	North (*n* = 2731)		South (*n* = 3641)		East (*n* = 5177)		West (*n* = 2572)		Central (*n* = 6425)		North Eastern (*n* = 806)	
	n	%	n	%	n	%	n	%	n	%	n	%
*Child characteristics*												
Sex of baby												
Male	1443	52.8	1880	51.7	2740	52.9	1292	50.2	3190	51.4	399	49.5
Female	1288	47.2	1761	48.4	2436	47.1	1280	49.8	3015	48.6	407	50.6
Age of child (months)												
0–2.9	1108	40.6	1467	40.3	2117	40.9	1054	41.0	2681	43.0	351	43.5
3–5.9	1623	59.4	2174	59.7	3060	59.1	1518	59.0	3525	57.0	455	56.5
Birth order												
First-born	1130	41.4	1606	4.1	1901	36.7	1074	41.8	2113	34.1	323	40.1
2nd-4th	1428	52.3	1994	54.7	2863	55.3	1438	55.9	3506	56.5	420	52.1
5 or more	173	6.3	41.0	1.1	413	8.0	60	2.3	587	9.5	63	7.8
Perceived size of baby												
Small	312	11.6	349	9.7	670	13.1	321	12.5	928	15.1	116	15.6
Average	2007	74.5	2235	61.9	3303	64.5	1670	65.3	4411	71.9	483	64.6
Large	374	13.9	1025	28.4	1145	22.4	568	22.2	797	13.0	148	19.8
Preceding birth interval												
No previous birth	1139	41.7	1630	44.8	1914	37.0	1081	42.0	2122	34.2	323	40.1
< 24 months	401	14.7	562	15.4	751	14.5	357	13.9	997	16.1	70	8.7
> 24 months	1191	43.6	1449	39.8	2511	48.5	1133	44.1	3087	49.7	412	51.1
*Maternal characteristics*												
Mother’s age												
15–24 years	154	6.1	341	9.4	714	13.8	243	9.4	317	4.9	94	11.6
25–34 years	2247	89.5	3211	88.2	4209	81.3	2259	87.8	5779	90.0	639	79.3
35–49 years	111	4.4	90	2.5	253	4.9	70	2.7	329	5.1	73	9.1
Mother’s education												
No education	758	27.8	358	9.8	1870	36.1	346	13.5	1987	32.0	152	18.8
Primary	363	13.3	258	7.1	751	14.5	293	11.4	943	15.2	140	17.3
Secondary and higher	1610	58.9	3025	83.1	2555	49.4	1932	75.1	3275	52.8	515	63.9
Mother’s religion												
Hindu	2074	76.0	2965	81.4	3885	75.0	2083	81.0	5209	83.9	380	47.2
Muslim	385	14.1	475	13.1	1098	21.2	342	13.3	959	15.5	269	33.4
Christianity and others	272	10.0	201	5.5	194	3.8	147	5.7	38	0.6	156	19.4
Type of caste or tribe												
Scheduled caste	718	26.3	841	23.1	1139	22.0	355	13.8	1408	22.7	67	8.3
Scheduled tribe	266	9.7	235	6.4	611	11.8	430	16.7	673	10.8	215	26.6
Other backward class	983	36.0	20	55.5	2179	42.1	836	32.5	3065	49.4	167	20.7
Others	764	28.0	20	15.0	1248	24.1	951	37.0	1060	17.1	357	44.4
*Family/household characteristics*												
Marital status												
Currently married	2721	99.7	3637	99.9	5153	99.7	2555	99.4	6172	99.5	799	99.3
Formerly married (divorced/separated/ widowed)	7	0.3	4	0.1	16	0.3	14	0.6	29	0.5	5	0.7
Household wealth index												
Poor	755	27.7	798	21.9	3826	73.9	732	28.5	3391	54.7	524	65.0
Middle	498	18.2	1025	28.1	746	14.4	587	22.8	1150	18.5	157	19.5
Rich	1478	54.1	1819	50.0	605	11.7	1253	48.7	1664	26.8	125	15.5
*Health service characteristics*												
Antenatal clinic visits												
None	308	11.3	263	7.2	1364	26.4	243	9.5	1037	16.7	112	13.9
1–3	1006	36.8	455	12.5	1821	35.2	448	17.4	2988	48.2	304	37.7
4+	1418	51.9	2923	80.3	1992	38.5	1880	73.1	2181	35.1	390	48.4
Place of delivery												
Home	300	11.0	109	3.0	1401	27.1	185	7.2	1412	22.8	239	29.6
Health facility	2431	89.0	3532	97.0	3775	72.9	2387	92.8	4793	77.2	567	70.4
Type of delivery assistance												
Health professional	2239	82.3	3244	89.2	3154	61.4	2060	80.6	3645	59.1	556	70.6
Traditional birth attendants	182	6.7	78	2.2	832	16.2	84	3.3	760	12.3	83	10.5
Other untrained personnel	300	11.0	316	8.7	1150	22.4	413	16.2	1765	28.6	149	18.9
Mode of delivery												
Vaginal	2291	83.9	2121	58.3	4497	86.9	1984	77.2	5488	88.4	667	82.8
Caesarean	440	16.1	1520	41.8	680	13.1	588	22.9	718	11.6	138	17.2
*Community-level factor*												
Residence												
Urban	878	32.2	1388	38.1	760	14.7	10,651	41.3	1350	21.8	111	13.7
Rural	1853	67.8	2253	61.9	4417	85.3	1510	58.7	4856	78.3	695	86.3

### Regional distribution of EBF and other breastfeeding (BF) practices

**Fig. 1:**
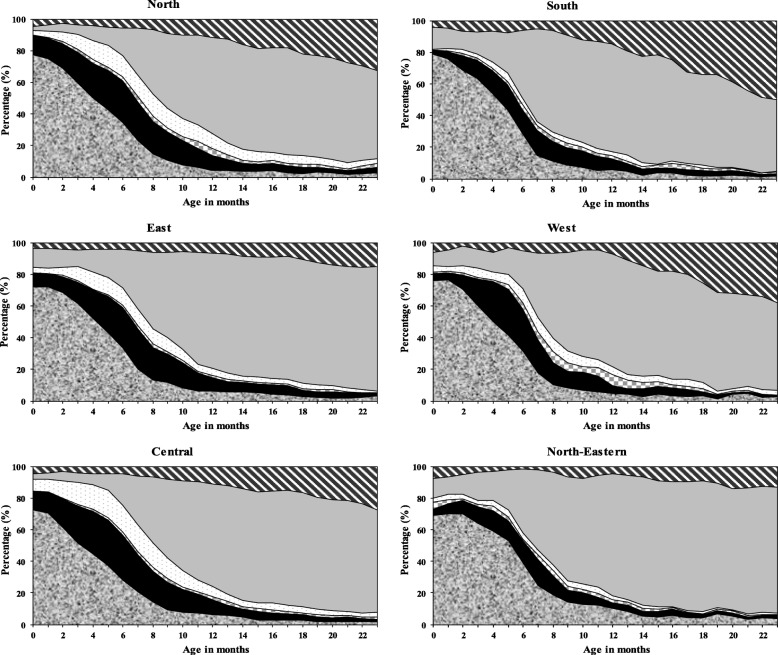
Regional distribution of exclusive breastfeeding (BF) and other infant and young child feeding practices by child age in India, 2015–2016 (NFHS-4)

As shown in Fig. 1, the prevalence of EBF among infants aged 0–5 months varied across the regions of India. EBF prevalence in infants aged 0–5 months was highest in the South (79.2%) and lowest in the North-Eastern region (68.0%). The EBF prevalence decreases as the infant grew older in the South (75.8% at 1 month of age, 63.6% at 3 months of age and 43.7% at 5 months) compared to the North-East region (70.0% at 1 month of age, 64.2% at 3 months and 54.0% at 5 months). Similarly, the proportion of children who received breast milk plus water, or breast milk plus water-based liquid or juice, breast milk plus other milk, or breast milk plus complementary foods from birth to 23 months of age varied substantially across Indian regions [Fig. 1].

### Regional determinants of exclusive breastfeeding

Across all regions, children in the 2nd–4th and five or more birth order categories (in the North, Central, and North-East region) were less likely to be exclusively breastfed compared to first-born children (Table 2). Mothers with secondary and higher level of education were less likely to exclusively breastfeed their infants compared to those with no education in Southern India. In contrast, mothers with a similar level of education were more likely to exclusively breastfeed their infants compared to those with no education in Central India.

**Table 2:** Factors associated with exclusive breastfeeding among infants aged 0–5 months by region in India, 2015–16 NFHS

Regions	North		South		East		West		Central		North-East	
Study variables	aOR (95% CI)	*P*-value	aOR (95% CI)	*P*-value	aOR (95% CI)	*P*-value	aOR (95% CI)	*P*-value	aOR (95% CI)	*P*-value	aOR (95% CI)	*P*-value
*Child factors*												
Sex of baby												
Male			*Ref.*									
Female			0.75(0.59, 0.95)	0.015								
Birth order												
First-born	*Ref.*		*Ref.*		*Ref.*		*Ref.*		*Ref.*		*Ref.*	
2nd-4th	0.71(0.60, 0.85)	< 0.001	0.56(0.45, 0.70)	< 0.001	0.70(0.59, 0.83)	< 0.001	0.46(0.34, 0.61)	< 0.001	0.80(0.71, 0.91)	< 0.001	0.50(0.39, 0.64)	< 0.001
5 or more	0.59(0.42, 0.83)	0.003	0.54(0.16, 1.83)	0.32	0.80(0.60, 1.08)	0.144	0.67(0.34, 1.31)	0.239	0.68(0.54, 0.85)	0.001	0.32(0.21, 0.48)	< 0.001
Perceived size of the baby												
Small			*Ref.*									
Average			0.74(0.50, 1.10)	0.137								
Large			0.53(0.35, 0.80)	0.003								
*Maternal factors*												
Mother’s education												
No education			*Ref.*						*Ref.*		*Ref.*	
Primary			1.11(0.66, 1.84)	0.701					1.13(0.96, 1.34)	0.145	1.13(0.96, 1.34)	0.145
Secondary and higher			0.59(0.41, 0.84)	0.003					1.24(1.07, 1.43)	0.004	1.24(1.07, 1.43)	0.004
Mother’s religion												
Hindu					*Ref.*				*Ref.*			
Muslim					0.96(0.76, 1.20)	0.703			0.75(0.63, 0.90)	0.001		
Christianity and others					0.55(0.36, 0.85)	0.006			1.26(0.68, 2.35)	0.459		
Type of caste or tribe												
Scheduled caste					*Ref.*		*Ref.*		*Ref.*			
Scheduled tribe					1.47(1.09, 1.99)	0.012	0.76(0.39, 1.48)	0.417	1.82(1.49, 2.22)	< 0.001		
Other backward class					0.82(0.67, 1.00)	0.049	0.85(0.50, 1.43)	0.531	1.00(0.86, 1.17)	0.969		
Others					0.95(0.73, 1.25)	0.729	0.56(0.32, 0.99)	0.046	0.83(0.68, 1.00)	0.055		
*Household factors*												
Marital status												
Currently married									*Ref.*			
Formerly married (divorced/separated/ widowed)									0.32(0.14, 0.70)	0.004		
Household wealth index												
Poor									*Ref.*			
Middle									0.89(0.77, 1.04)	0.155		
Rich									0.69(0.59, 0.81)	< 0.001		
*Health service factors*												
Antenatal clinic visits												
None	*Ref.*											
1–3	1.13(0.87, 1.48)	0.361										
4+	1.34(1.02, 1.76)	0.035										
Type of delivery assistance												
Health professional									*Ref.*		*Ref.*	
Traditional birth attendant									0.83(0.69, 0.99)	0.043	0.73(0.50, 1.06)	0.100
Other untrained personnel									0.89(0.78, 1.01)	0.071	1.49(1.08, 2.06)	0.016
Mode of delivery												
Vaginal											*Ref.*	
Caesarean section											1.72(1.24, 2.40)	0.001
*Community-level factor*												
Place of residence												
Urban							*Ref.*					
Rural							0.71(0.51, 0.99)	0.045				

Statistically significant (using p-value < 0.05 and confidence intervals) study factors from multivariable models are shown. In the model of child factors, adjustments were made for maternal, household, health service and community factors. A similar approach was used for maternal, household, health service and community factors that adjusted for respective factors in multivariable models. *aOR* adjusted odds ratio, *ref*. reference category

Lower EBF practice was observed among mothers with Christian and another religious background in Eastern India and among mothers of Muslim background in Central region. Mothers who belonged to the ‘Scheduled tribe’ had higher odds of practising EBF in East and North-East regions of India. In contrast, mothers from ‘Other backward classes’ in the East region were less likely to practice EBF (Table 2). In Southern India, children were less likely to be exclusively breastfed if they were female and were perceived to be born large compared to being male and those perceived to be small. In North-East India, EBF practice was significantly lower among mothers who were formerly married and higher among mothers whose infants were born via caesarean section compared to currently married mothers and those whose infants were born vaginally (Table 2).

In North India, infants born to mothers who had four or more antenatal visits were more likely to be exclusively breastfed compared to those whose mothers had no antenatal visit. Infants whose mothers resided in rural areas of Western India were less likely to be exclusively breastfed compared to their counterparts in urban areas. In Central India, children born to mothers from rich households were less likely to be exclusively breastfed compared to mothers who were from poor households (Table 2).

## Discussion

Our study showed that substantial variations exist for the prevalence of EBF and other childhood feeding practices across the regions of India, demonstrating the socioeconomic, religious, cultural and geographical diversity in the country. Mothers who resided in Southern India had the highest EBF prevalence, while those in the North-East had the lowest prevalence. EBF prevalence decreased with infant age, declining faster in the South (43.7% at 5 months) compared to the North-East region (54.0% at 5 months). Similarly, this study found that the factors associated with EBF also varied widely across regional lines, where higher birth order was the only common factor associated with non-EBF in all regions of India.

In India, an estimated 0.9 million under-five deaths were reported in 2016 [7]. Of those deaths, approximately 50% occurred in the neonatal period, a time when EBF has been shown to significantly improve child survival [27]. Our study indicated that EBF prevalence was lowest in the North-Eastern region, while in all regions, EBF prevalence decreased with the infant age, with the prevalence falling below 50% around the age of five months. These suboptimal EBF figures could be attributed to inadequate healthcare financing, planning and strategic actions on infant feeding [28]. At the community level in India, common traditional infant feeding practices associated with the early introduction of water and other water-based fluids before six months of age have also hindered interventions to promote EBF [29, 30]. The establishment of national and subnational policy, programme and coordination for IYCF practices, as well as allocating special funds for implementation of those interventions remain key strategies for increasing India’s EBF [28].

The present study showed that children of higher birth order (2nd or more) were less likely to be exclusively breastfed compared to first-born children in all regions. This result is inconsistent with evidence from Nigeria [26, 31], Tanzania [32] and regional areas of Sri Lanka [33] which found no association between the birth order of the child and EBF. In the Sri Lankan study, however, second born babies had a higher EBF rate compared to first-born babies, and the rate dropped further from the third baby [33]. In comparison to countries (such as Sri Lanka and Tanzania) with established national and subnational policy, programme and coordination for IYCF practices [34, 35], India’s national and subnational IYCF strategy, as well as breastfeeding training for front-line health workers and support for new mothers, is inadequate [28]. This may be one of the reasons as to why subsequent births were less likely to exclusively receive only breast milk in the first six months of life. Additionally, a study from India reported that undernutrition was associated with first-born babies compared to subsequent births, and the burden increases further with increasing birth order [36], highlighting the issue of a lack of coordinated action for IYCF programmes in the country.

Improved maternal education is one of the single most important measures to improve not only child nutrition and survival but also maternal health and household social-emotional interaction [37, 38]. Our study suggests that higher maternal education was associated with non-EBF in the Southern region, but the opposite association was observed in the Central region of India, where higher maternal education was associated with EBF. Additionally, mothers from more wealthy households were less likely to engage in EBF compared to those from poorer households in Central India. Higher maternal education is linked with increased opportunity for professional employment and subsequently improved household income [39]. Mothers who are employed are less likely to engage in EBF, especially when they work in environments that provide limited opportunities for optimal breastfeeding [39, 40]. In comparison to Central India, the Southern region has a higher literacy rate [10], better economic indices and opportunities for employment [41]. These factors may account for the observed differences in EBF among mothers from those regions.

Notably, the Government of India has recently taken actions to improve maternal and child health, including EBF. These efforts include the introduction of various schemes (e.g. Pradhan Mantri Matru Vandana Yojana, PMMVY) [42] and recent amendments to the Maternity Benefit Act, 1961 [henceforth, Maternity Benefit (Amendment) Act, 2017]. The amendment protects women’s employment, and women’s and children’s well-being during maternity, with paid absence and related benefits. It also increased the maternity leave from 12 weeks to 26 weeks and makes the provision of crèche facility mandatory for every company employing more than 50 employees, with the specific target of improving the EBF [43].

Furthermore, improving EBF participation in India would also require increased girl child education as articulated in the Sustainable Development Goals [44] and the full implementation of the India World Breastfeeding Trends Initiative (WBTi) recommendations [28]. These include the establishment of the Baby Friendly Hospital Initiative (BFHI) centres and the provision of information support for breastfeeding, as well as strengthening monitoring and evaluation systems. In 2016, the Government of India introduced the Mothers Absolute Affection (MAA) programme, with a focus to intensify efforts towards the promotion, protection and support of optimal breastfeeding across regions of the country. The programme aims to i) generate breastfeeding activities and provide an enabling environment for pregnant and lactating mothers, family member and community; ii) intensify and support breastfeeding-related activities in public health facilities through facility- and community-based healthcare workers; and iii) recognise and provide additional support to healthcare facilities with improved breastfeeding outcomes [45]. While this initiative provides a good opportunity for Indian mothers to increase breastfeeding for their children, efforts such as revitalising the country’s BFHI remain a key priority [28].

Consistent with past reports [25, 26, 32], our study indicated that frequent (≥4) antenatal visits were associated with EBF among mothers in Northern India. The WHO recommends that pregnant women should attend at least four antenatal visits to create opportunities for risk identification; prevention and management of pregnancy-related and/or comorbidities; and health education and health promotion, including the provision of EBF information [46]. More recently, the Government of India has introduced various maternal health schemes (e.g. Pradhan Mantri Surakshit Matritva Abhiyan, PMSMA; PMMVY and LaQshya programmes). These interventions aim to improve antenatal care uptake and ensure quality care during pregnancy, birthing and the immediate post-partum period for women and their newborn [42, 47]. While these initiatives are needed and well-deserved to improve not only maternal health but also newborn care (including EBF), the establishment and integration of IYCF programme interventions with the programmes are also critical to improving EBF and subsequent child survival in India.

In the present study, other significant factors associated with non-EBF included residence in rural areas in Western India; female gender and perceived large babies in Southern India; practising Christianity and other religious backgrounds in Eastern India and being of Muslim background in North-Eastern regions. In contrast, belonging to the ‘Scheduled tribe’ was associated with EBF in East and North-East regions of India, while mothers from ‘Other backward class’ had lower odds of EBF in Eastern India. Evidence from many developing countries [11, 32, 48–50] has shown that the determinants of EBF practice are multifaceted, and therefore, interventions aimed at increasing EBF rates would require a multipronged approach that considers the specific environment in which mothers raise their children. The India WBTi recommended the allocation of special funds for IYCF policy and programmes implementation as a major strategy for improving IYCF in the country [28]. Importantly, the Indian government is already dedicating funds to promote IYCF as part of various national programmes, including the Home-Based Newborn Care, the Home Based Young Child Care and the Integrated Child Development Services [42, 47]. However, whether these interventions are targeted at high-risk populations to increase EBF participation among Indian women remains unclear. Future studies that evaluate the positive impacts of these policy interventions in the context of EBF may be warranted to inform refinement of future programmes.

### Study limitations and strengths

The study has several methodological limitations that should be considered when interpreting the results. First, we used cross-sectional data, and this makes the articulation of a clear temporal association between the study factors and EBF difficult. Second, the DHS data used for this study were collected through self-report, and this is a source of recall bias which may have either underestimated or overestimated the relationship between the study factors and EBF. Finally, we were unable to measure all possible variables and/or contextual factors that could potentially affect EBF in India, including partner support, a cultural belief system for infant feeding and health professional’s knowledge of EBF. The lack of assessment of these unmeasured factors may have affected our results. Despite these methodological limitations, the study has strengths. First, we used data that had high response rates (from 94.0 to 99.6% across the states of India) suggesting that the potential effect of selection bias is unlikely to be present in our study findings. Second, the India DHS data were collected by skilled personnel using standardised questionnaires which ensured that the data collected were consistent across the states and territories of India. Lastly, our study provided evidence on important modifiable factors associated with EBF in the world’s second largest populations to help nutrition experts in the country advocate for effective policies and intervention services to improve EBF in India.

## Conclusion

The present study suggests that there were considerable variations in the prevalence of EBF and other IYCF practices across the regions of India. Southern India had the highest EBF prevalence, while the North-East had the lowest prevalence. The determinants of EBF also varied across regional lines, where higher birth order was the only common factor associated with non-EBF in all regions. Key modifiable determinants of non-EBF included higher maternal education in the South and belonging to rich households in Central India. Efforts to improve EBF in India would require a multipronged approach, where political will and dedicated funds for those public health actions and the establishment of IYCF policy and programmes interventions remain core priorities.

## Additional file

Additional file 1: Univariate analyses of factors associated with exclusive breastfeeding among infants aged 0–5 months by region in India, 2015–16 NFHS (PDF 212 kb)

## Abbreviations

DHS: Demographic and health survey
EBF: Exclusive breastfeeding
IIPS: International Institute for Population Sciences
NFHS: National Family and Health Survey
