# Correction to: Regional prevalence and determinants of exclusive breastfeeding in India

**DOI:** 10.1186/s13006-019-0220-2

**Published:** 2019-06-04

**Authors:** Felix Akpojene Ogbo, Mansi Vijaybhai Dhami, Akorede O. Awosemo, Bolajoko O. Olusanya, Jacob Olusanya, Uchechukwu L. Osuagwu, Pramesh Raj Ghimire, Andrew Page, Kingsley E. Agho

**Affiliations:** 1 Translational Health Research Institute (THRI), School of Medicine, Western Sydney University, Campbelltown Campus, Locked Bag 1797, Penrith, NSW 2571 Australia; 2 Prescot Specialist Medical Centre, Welfare Quarters, Makurdi, Benue State Nigeria; 3 Centre for Healthy Start Initiative, 286A Corporation Drive, Dolphin Estate, Ikoyi, Lagos, Nigeria; 4 School of Medicine | Diabetes Obesity and Metabolism Translational Research Unit (DOMTRU), Macarthur Clinical School, Parkside Crescent, Campbelltown, NSW 2560 Australia; 5 School of Science and Health, Western Sydney University, Campbelltown Campus, Locked Bag 1797, Penrith, NSW 2571 Australia

**Correction to: International Breastfeeding Journal (2019) 14:20**

**DOI: 10.1186/s13006-019-0214-0**

Please note that in the original article [1], Fig. 1 was originally published with its figure legend erroneously omitted.

**Fig. 1:**
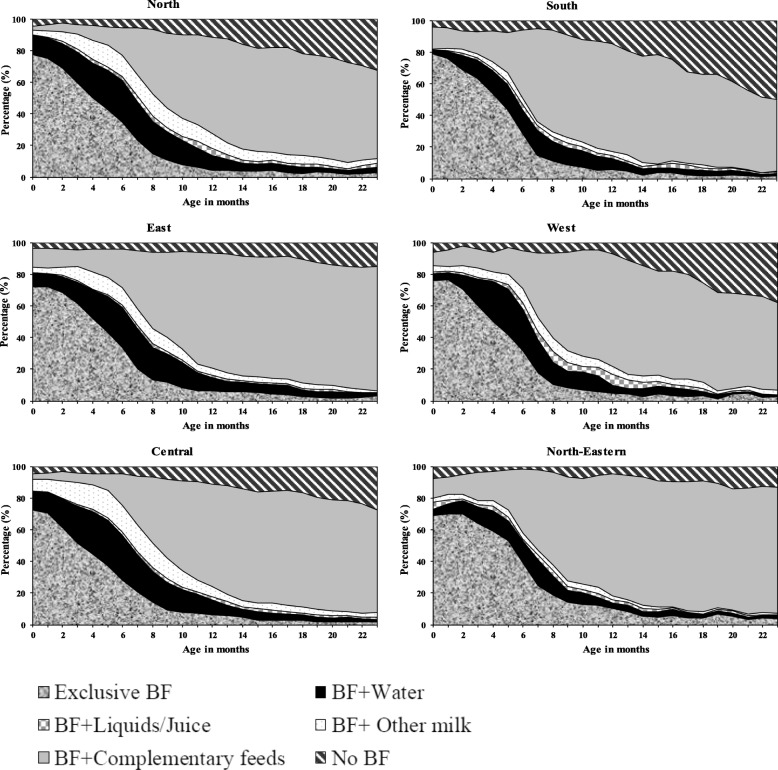
Regional distribution of exclusive breastfeeding (BF) and other infant and young child feeding practices by child age in India, 2015–2016 (NFHS-4)

Please see below for the correct version of Fig. 1 (with its legend included):

The figure has been corrected in the original article.

The publisher apologizes for this processing error.
