# Supplementary material for: Regional prevalence and determinants of exclusive breastfeeding in India
Source: Int Breastfeed J. 2019 May 16;14:20. doi: 10.1186/s13006-019-0214-0 (PMC6524302; doi:10.1186/s13006-019-0214-0)
Supplement: Supplementary file 1 — Univariate analyses of factors associated with exclusive breastfeeding among infants aged 0–5 months by region in India, 2015–16 NFHS (PDF 212 kb) [file 13006_2019_214_MOESM1_ESM.pdf]

Additional file 1:

	North		South		East		West		Central		North-East	
**Study variables**	**COR(95% CI)**	**P-Value**	**OR(95% CI)**	**P-Value**	**OR(95% CI)**	**P-Value**	**OR(95% CI)**	**P-Value**	**OR(95% CI)**	**P-Value**	**OR(95% CI)**	**P-Value**
Child factors												
**Sex of baby**												
Male	*Ref.*		*Ref.*		*Ref.*		*Ref.*		*Ref.*		*Ref.*	
Female	0.94(0.80, 1.11**)**	0.513	0.73(0.58, 0.91)	0.006	0.99(0.85, 1.16)	0.987	0.78(0.58, 1.05)	0.104	0.96(0.86, 1.06)	0.466	1.20(0.95, 1.53)	0.122
**Child age**												
0-2.9 months	*Ref.*		*Ref.*		*Ref.*		*Ref.*		*Ref.*		*Ref.*	
3-5.9 months	0.36(0.30, 0.43)	<0.001	0.40(0.32, 0.51)	0.001	0.40(0.34, 0.47)	<0.001	0.32(0.22, 0.46)	<0.001	0.39(0.35, 0.44)	<0.001	0.53(0.43, 0.66)	<0.001
**Birth order**												
First-born	*Ref.*		*Ref.*		*Ref.*		*Ref.*		*Ref.*		*Ref.*	
2nd-4th	0.73(0.62,0. 87)	<0.001	0.60(0.49, 0.75)	<0.001	0.69(0.58, 0.82)	<0.001	0.46(0.34, 0.61)	<0.001	0.80(0.71, 0.90)	<0.001	0.49(0.38, 0.62)	<0.001
5 or more	0.58(0.41, 0.83)	0.003	0.57(0.37, 0.88)	0.438	0.78(0.58, 1.04)	0.096	0.74(0.38, 1.43)	0.374	0.63(0.51, 0.78)	<0.001	0.33(0.22, 0.50)	<0.001
**Perceived size of the baby**												
Small	*Ref.*		*Ref.*		*Ref.*		*Ref.*		*Ref.*		*Ref.*	
Average	1.18(0.92, 1.52)	0.171	0.77(0.51, 1.16)	0.218	0.95(0.76, 1.20)	0.717	0.89(0.58, 1.38)	0.62	1.05(0.91, 1.23)	0.454	0.93(0.66, 1.30)	0.682
Large	1.14(0.83, 1.56)	0.84	0.53(0.35, 0.80)	0.011	0.89(0.67, 1.19)	0.458	0.79(0.48, 1.30)	0.361	1.14(0.92, 1.40)	0.216	1.00(0.66, 1.51)	0.988
**Preceeding birth interval**												
No previous birth	*Ref.*		*Ref.*		*Ref.*		*Ref.*		*Ref.*		*Ref.*	
<24 months	0.74(0.58, 0.95)	0.019	0.55(0.39, 0.76)	<0.001	0.73(0.57, 0.92)	0.01	0.36(0.22, 0.59)	<0.001	0.76(0.65, 0.89)	0.001	0.39(0.26, 0.58)	<0.001
>24 months	0.72(0.60, 0.86)	<0.001	0.65(0.52, 0.83)	<0.001	0.70(0.58, 0.84)	<0.001	0.51(0.37, 0.71)	<0.001	0.78(0.69, 0.88)	<0.001	0.47(0.37, 0.61)	<0.001
Maternal factors												
**Mother's age**												
15-24 years	*Ref.*		*Ref.*		*Ref.*		*Ref.*		*Ref.*		*Ref.*	
25-34 years	0.69(0.48, 0.99)	0.049	0.62(0.43, 0.90)	0.013	0.80(0.62, 1.03)	0.098	0.77(0.46, 1.27)	0.316	0.87(0.69, 1.10)	0.274	0.58(0.40, 0.85)	0.005
35-49 years	0.55(0.32, 0.93)	0.027	0.79(0.36, 1.73)	0.558	0.65(0.44, 0.97)	0.038	0.76(0.32, 1.79)	0.539	0.80(0.57, 1.11)	0.193	0.65(0.40, 1.05)	0.083

**Mother's education**												
No education	*Ref.*		*Ref.*		*Ref.*		*Ref.*		*Ref.*		*Ref.*	
Primary	0.98(0.74, 1.29)	0.896	0.93(0.57, 1.52)	0.801	0.92(0.73, 1.17)	0.526	0.76(0.43, 1.35)	0.362	1.15(0.98, 1.36)	0.074	0.71(0.50, 1.01)	0.058
Secondary and higher	1.15(0.94, 1.40)	0.155	0.59(0.41, 0.83)	0.003	0.94(0.80, 1.11)	0.52	1.05(0.70, 1.58)	0.781	1.19(1.06, 1.35)	0.004	1.14(0.86, 1.52)	0.339
**Mother’s religion**												
Hindu	*Ref.*		*Ref.*		*Ref.*		*Ref.*		*Ref.*		*Ref.*	
Muslim	1.07(0.85, 1.36)	0.526	0.99(0.73, 1.36)	0.992	0.91(0.74, 1.13)	0.41	0.55(0.36, 0.85)	0.007	0.57(0.48, 0.67)	<0.001	0.71(0.63, 0.90)	0.023
Christianity and others	1.09(0.84, 1.43)	0.493	0.85(0.51, 1.41)	0.545	0.73(0.49, 1.10)	0.142	1.91(0.89, 4.07)	0.093	1.23(0.65, 2.31)	0.511	053(0.68, 2.35)	<0.001
**Type of Caste or tribe**												
Scheduled caste	*Ref.*		*Ref.*		*Ref.*		*Ref.*		*Ref.*		*Ref.*	
Scheduled tribe	0.77(0.57, 1.03)	0.085	1.08(0.67, 1.76)	0.735	1.33(1.01, 1.74)	0.037	0.80(0.42, 1.51)	0.5	1.97(1.62, 2.40)	<0.001	0.95(0.63, 1.41)	0.804.
Other backward class	0.88(0.72, 1.08)	0.254	0.94(0.72, 1.21)	0.647	0.79(0.65, 0.97)	0.026	0.90(0.56, 1.45)	0.684	0.92(0.79, 1.06)	0.267	1.68(1.05, 2.68)	0.027
Others	0.90(0.73, 1.13)	0.398	0.97(0.68, 1.39)	0.881	0.93(0.72, 1.20)	0.611	0.56(0.33, 0.94)	0.031	0.72(0.60, 0.87)	0.001	1.22(0.80, 1.85)	0.34
Household factors												
**Marital status**												
Currently married	*Ref.*		*Ref.*		*Ref.*		*Ref.*		*Ref.*		*Ref.*	
Formerly married (div/separated/widow)	3.01(0.78, 11.54)	0.108	1.25(0.09, 15.71)	0.862	0.50(0.16, 1.57)	0.241	0.35(0.06, 2.01)	0.245	0.33(0.14, 0.74)	0.008	0.49(0.21, 1.14)	0.099
**Household wealth Index**												
Poor	*Ref.*		*Ref.*		*Ref.*		*Ref.*		*Ref.*		*Ref.*	
Middle	1.17(0.92, 1.47)	0.182	0.71(0.53, 0.95)	0.021	0.89(0.72,1.11)	0.328	0.91(0.62, 1.33)	0.629	0.86(0.75, 1.00)	0.054	0.90(0.68. 1.19)	0.5
Rich	1.19(0.99, 1.43)	0.059	0.66(0.51, 0.86)	0.003	0.93(0.70,1.23)	0.626	0.89(0.64, 1.26)	0.534	0.71(0.62, 0.81)	<0.001	1.35(0.99, 1.83)	0.053
**Reads newspaper or magazine**												
Not at all	*Ref.*		*Ref.*		*Ref.*		*Ref.*		*Ref.*		*Ref.*	
Yes	1.2(1.01, 1.42)	0.034	0.92(0.74, 1.14)	0.481	0.86(0.72, 1.03)	0.107	0.98(0.71, 1.34)	0.92	1.02(0.91, 1.16)	0.636	0.70(0.53,0.91)	0.01
**Listens to radio**												
Not at all	*Ref.*		*Ref.*		*Ref.*		*Ref.*		*Ref.*		*Ref.*	
Yes	1.04(0.82, 1.34)	0.699	0.72(0.53,0.96)	0.028	0.85(0.68, 1.05)	0.14	0.83(0.42, 1.66)	0.612	1.12(0.95, 1.32)	0.168	1.43(1.05,1.95)	0.02

**Watched television**												
Not at all	*Ref.*		*Ref.*		*Ref.*		*Ref.*		*Ref.*		*Ref.*	
Yes	1.18(0.98, 1.42)	0.078	0.89(0.58, 1.36)	0.609	0.99(0.84, 1.16)	0.95	1.07(0.71, 1.62)	0.72	1.01(0.90, 1.13)	0.798	1.27(0.99, 1.63)	0.051
Health service factors												
**Antenatal clinic visits**												
None	*Ref.*		*Ref.*		*Ref.*		*Ref.*		*Ref.*		*Ref.*	
1-Mar	1.15(0.88, 1.51)	0.291	1.50(0.90, 2.51)	0.114	1.1(0.91, 1.31)	0.298	1.31(0.79, 2.16)	0.289	0.92(0.79, 1.08)	0.351	1.26(0.90, 1.75)	0.166
4+	1.43(1.10, 1.88)	0.008	1.59(1.02, 2.48)	0.037	1.15(0.94, 1.41)	0.16	1.16(0.74, 1.81)	0.5	1.07(0.91, 1.27)	0.358	1.49(1.08, 2.05)	0.015
**Place of delivery**												
Home	*Ref.*		*Ref.*		*Ref.*		*Ref.*		*Ref.*		*Ref.*	
Health Facility	1.28(0.98, 1.67)	0.067	0.85(0.38, 1.90)	0.709	1.01(0.85, 1.19)	0.883	0.77(0.49, 1.21)	0.275	1.18(1.04, 1.35)	0.01	1.22(0.96, 1.54)	0.096
**Type of delivery assistance**												
Health professional	*Ref.*		*Ref.*		*Ref.*		*Ref.*		*Ref.*		*Ref.*	
Traditional birth attendant	0.75(0.53, 1.05)	0.1	2.25(1.09, 4.64)	0.027	1.05(0.85, 1.28)	0.62	1.16(0.62, 2.16)	0.627	0.83(0.69, 0.99)	0.035	0.55(0.38, 0.79)	0.001
Other untrained	0.98(0.75, 1.27)	0.892	1.03(0.72, 1.47)	0.853	1.12(0.93, 1.35)	0.204	1.01(0.57, 1.78)	0.958	0.89(0.78, 1.01)	0.058	1.19(0.88, 1.62)	0.253
**Mode of delivery**												
Vaginal	*Ref.*		*Ref.*		*Ref.*		*Ref.*		*Ref.*		*Ref.*	
Caesarean section	0.99(0.80, 1.23)	0.984	1.08(0.87, 1.35)	0.448	1.05(0.79, 1.40)	0.36	1.56(1.08, 2.26)	0.017	0.80(0.69, 0.93)	0.004	1.82(1.33, 2.49)	<0.001
Community-level factor												
**Place of residence**												
Urban	*Ref.*		*Ref.*		*Ref.*		*Ref.*		*Ref.*		*Ref.*	
Rural	0.88(0.72, 1.06)	0.203	0.78(0.62, 0.98)	0.036	1.05(0.79, 1.40)	0.719	0.71(0.51, 0.99)	0.045	0.80(0.69, 0.93)	0.004	1.07(0.81, 143)	0.601

COR: crude odds ratio; 95% CI: 95% confidence interval; ref: reference
